# Ground-up generation of periodic slab models of dehydroxylated amorphous silica of varying roughness†

**DOI:** 10.1039/d5cp01570g

**Published:** 2025-06-26

**Authors:** Mas P. Klein, Evgeny A. Pidko, Alexander A. Kolganov

**Affiliations:** a Inorganic Systems Engineering, Department of Chemical Engineering, Faculty of Applied Sciences, Delft University of Technology Van der Maasweg 9 2629 HZ Delft The Netherlands a.a.kolganov@tudelft.nl

## Abstract

Simulation and systematic analysis of the surfaces of amorphous materials is a challenge for computational chemistry. For example, silica has found widespread industrial use as an adsorbent and catalyst support but available models for use with periodic DFT are limited in variety and representativeness of realistic materials. Herein we present a generic approach for the systematic construction of ensembles of amorphous materials surface models with varied roughness and termination characteristics. The power of the approach is shown with silica as the representative example. By combining MD simulations and Fourier-series-based randomization, bulk amorphous silica was modeled and cleaved to produce surfaces with systematically varied roughness and surface saturation. An automated saturation procedure resulted in surface models with silanol densities typical of high-temperature activation protocols in the range 0.35–2.00 OH nm^−2^, in excellent agreement with the experimental data on surface chemistry of dehydroxylated silica materials.

## Abstract 2

The generic approach for the systematic construction of ensembles of amorphous materials surface models with varied roughness and termination characteristics is presented.

## Graphical abstract

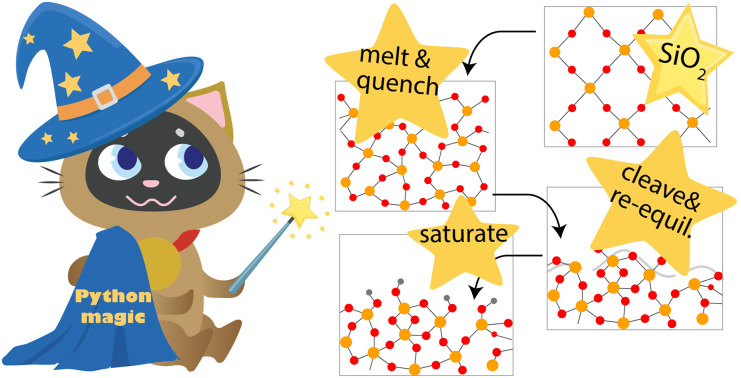

## 1 Introduction

The knowledge of the molecular structure of materials is critical for understanding and predicting their properties through computational modeling. For crystalline materials, their regular, periodic structures simplify model construction.^1^ However, many widely used materials lack this long-range periodic order,^2^ such as amorphous silica (a-SiO_2_), which finds diverse applications, including pharmaceuticals,^3,4^ optics,^5^ and catalysis.^6–8^

**Fig. 1:**
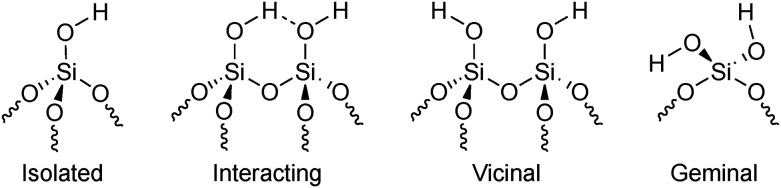
Different types of silanol groups found on the surface of amorphous silica.

High-temperature (*ca.* 700 °C) dehydroxylation *in vacuo* is a common approach to reduce the heterogeneity of reactive sites on silica surfaces and produce model support materials well-suited for surface organometallic chemistry applications^9–11^ and the generation of single-site catalysts.^6,12^ For a-SiO_2_, the resulting support can be referred to as SiO_2−700_. This treatment results in surfaces with silanol group (Fig. 1) concentrations of *ca.* 1.15 OH nm^−2^ (ref. 13 and 14) with a still debatable distribution of types.^1^ The results of Zhuravlev^13^ suggest that after high-temperature dehydroxylation, silica surface features exclusively isolated (90%) and geminal (10%) silanols, while the study by Fleischman and Scott^15^ presented experimental evidence of the formation of also vicinal SiOH species (Fig. 1). Beyond the types of silanol groups present, the precise local structures and catalytic roles of the diverse sites on amorphous surfaces are also often unknown.^16–18^

Computational modeling offers an opportunity to shed light on the structure and reactivity of such surface species. Modern approaches for constructing atomistic models of amorphous materials either focus on representing the local environment of the specific surface sites with finite cluster models^4,19–23^ or approximate the amorphous surface with distorted periodic slab models.^14,24–26^ While cluster models allow for detailed and highly accurate computations of local chemical behavior, they generally lack the high strain expected in highly dehydroxylated silica and a means of accounting for the inherent heterogeneity of the surface. Periodic slab models do not fall victim to these issues and provide a basis for systematically exploring the varied chemical environments of amorphous materials.

**Fig. 2:**
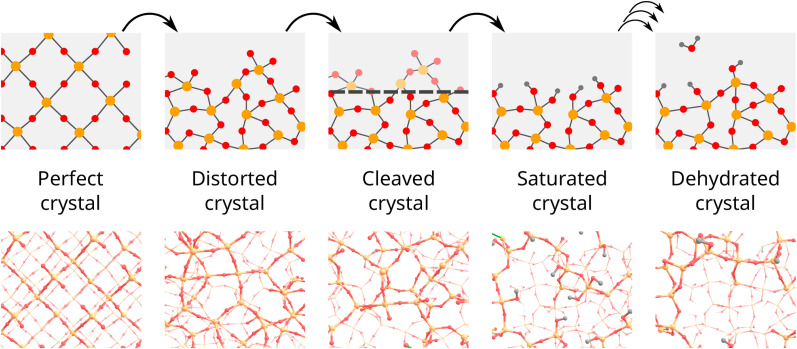
Overview of the general procedure commonly used for creating dehydroxylated periodic slab models of amorphous silica.

Fig. 2 schematically illustrates the common procedure for the construction of periodic slab surface models of amorphous materials (*e.g.* SiO_2−700_). First, the crystal structure of SiO_2_ (usually β-cristobalite) undergoes distortion *via* force-field MD simulation of melting and cooling processes. Next, a surface is created by cleaving the distorted structure with a plane at a fixed *z*-coordinate. The resulting dangling bonds are saturated by silanol groups, followed by successive condensation of neighboring SiOH pairs to ensure the overall charge neutrality and yield the dehydroxylated surface model.

This protocol results in relatively flat surface models^14,24,25,27^ and it does not allow for systematic variation and exploration of surface roughness, which plays a decisive role in surface properties^28,29^ and reactivity^30,31^ of a-SiO_2_. The roughness of the resulting model can be increased through quicker rates of cooling, although to a relatively limited extent.^32^ Nguyen and Laird^33^ proposed using a stochastic Fourier series in place of a flat plane at the cleaving step and showed that through their method, the roughness could effectively be controlled.

The surface resulting from the saturation step generally has silanol concentrations between 6 and 8 OH nm^−2^.^14,24,25,27^ For the reduction of silanol concentration to the dehydrated surface, the condensation step is ideally accompanied by relaxation and evaluation of the surface energetics. The consequences of a given condensation on surface stabilities cannot be known *a priori*,^14^ leading to noticeable computational and manual effort. Alternative approaches based on the addition of SiOH until the desired density is achieved have been proposed, efficient enough to produce a large number of models.^32,33^ However, the implementations may result in charged surfaces^33^ or remaining dangling bonds.^32,33^

Herein we present a method for the systematic generation of surfaces of amorphous materials enabling systematic variation of surface roughness and algorithmic surface saturation. The method is demonstrated for a-SiO_2_, for which a comprehensive set of models was generated. The resulting silanol concentrations and compositions were evaluated and compared to experiment and previously created models. To further validate models, atomistic thermodynamics was used to compute stability diagrams over temperature and *P*_H_2_O_ for three select surfaces functionalized with additional silanol groups.

## 2. Methods

**Fig. 3:**
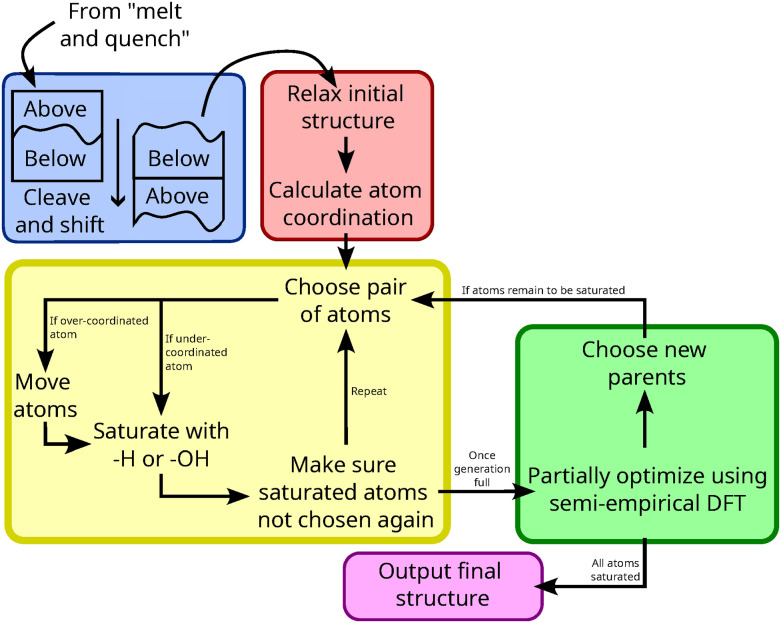
General overview of the method implemented to create surfaces and saturate dangling bonds of the models.

A model of bulk a-SiO_2_ was generated using the classical molecular dynamics carried out using the Large scale Atomic/Molecular Massively Parallel Simulator (LAMMPS)^34^ software package. To describe the interaction between atoms the forcefield of van Beest, Kramer, and van Santen (BKS)^35^ was used overlayed with the Lennard-Jones potential described by Wimalasiri *et al.*^32^ (parameters found in ESI† Table S1). An Ewald summation^36^ to a tolerance of 10^−4^ was used to account for long-range coulomb interactions. Using a time step of 0.5 fs, a 3 × 3 × 2 supercell of β-cristobalite^37^ (144 units of SiO_2_) was heated to 8000 K at constant pressure (1 atm) and subsequently cooled to 298 K at a rate of 1 K ps^−1^. This resulting bulk silica sample was heated to 4000 K and allowed to equilibrate for 200 ps then cooled to 298 K at a rate of 1 K ps^−1^. During these simulations, the *x* and *y* dimensions were held constant at 21.5 Å to simplify analysis. To control temperature and pressure, a Berendsen thermostat (damping constant of 0.1 ps) and barostat^38^ (damping constant of 0.1 ps, modulus of 360 000 atm) were employed. The resulting amorphized bulk was cleaved using the method of Nguyen and Laird^33^ using 10 different stochastic Fourier series with roughness being controlled through the empirical parameter *α*, which is essentially an attenuation factor controlling the decay of Fourier coefficients based on frequency magnitude. In simple terms, lower values of *α* result in surfaces of higher roughness, on average. For more details, the reader is referred to the work of Nguyen and Laird.^33^ For this study, *α* 's equal to 0.01, 0.02, 0.03, 0.04, 0.05, and 0.1 were used, resulting in a total of 60 different structures. To cleave the surface, atoms were not removed but rather shifted downwards by the *z* length of the simulation box, schematically shown in Fig. 3 (blue). Each structure was subsequently re-equilibrated for 2 ns at 298 K under an NVT ensemble with a 15 Å vacuum in the *z*-dimension.

The saturation of models was achieved through a genetic algorithm, summarized in Fig. 3. The initial, dry, structure was relaxed and dangling bonds were saturated in a step-wise manner. At each step, a single H_2_O molecule was added to the structure which was then relaxed. 10 possible structures were considered per step and partially optimized, after which the one of lowest electronic energy was carried forward. This continued until there were no remaining dangling bonds as they are uncommon at the high-temperature activation conditions (low humidity, 700 °C).^39^ Si and O atoms were considered bound to each other if they were within 2 Å of each other. This assumption is based on a geometric constraint that atoms are in contact if they are closer than 62% of the sum of their van der Waals radii.^40^ If necessary, dangling atoms were induced by shifting a Si atom within 2.0 Å of a 3-coordinate oxygen defect (^3^O). To achieve higher silanol densities, the models were functionalized through probing for surface atoms, as described in ref. 32, and water molecules were added across Si–O bonds, which were selected at random. Probing was carried out through splitting the *x*–*y* plane into a square grid with a spacing of 0.15 Å between points and a probe of 1.4 Å descending from the vacuum slab until contact is made with an atom. Contact was determined when the radius of the probe intersects the contact radius of an atom, defined as 1.04 Å for Si atoms and 0.76 Å for O atoms.

**Fig. 4:**
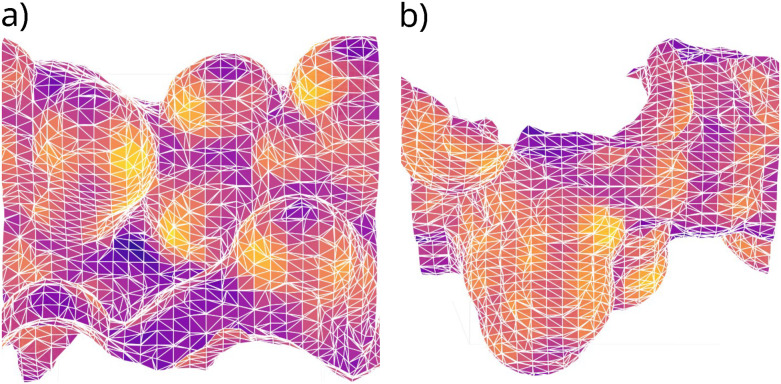
Representation of the meshes generated used to estimate surface areas of a model separated into the (a) “top” of the model and (b) “bottom” of the model.

The silanol density of the given sample can be assessed experimentally through physisorption of liquid Kr or N_2_.^13,14^ Taking inspiration from this, the solvent-accessible surface area for Kr was calculated as to estimate the surface area of generated models. To get this estimate, a polygon mesh of the structure was generated, like that shown in Fig. 4, using the marching cubes algorithm^41^ as implemented in scikit-image.^42^ There are two independent surfaces in the slab model. Both surfaces could be a valid model, so both sides were analyzed as individual data points. The surface area was taken as the sum of the area of all tetrahedrons that are part of the mesh. Starting from the top-most and bottom-most vertices of the mesh, the polygons belonging to either surface of the model were identified through a breadth-first search for all connecting vertices. The roughness of the model was calculated as the mean-square deviation of these vertices. To identify which silanol groups belong to which side of the model, *k*-means clustering^43^ based on the *z*-coordinate of the H atoms was used. It was assumed that there would be two distinct clusters corresponding to each side of the model and that silanol groups would find themselves close to the surfaces of the model.

For the entirety of the algorithm outlined in Fig. 3, structure relaxations were performed using the semi-empirical SCC-DFTB^44^ method as implemented in CP2K.^45^ The parameter set of Guimarães *et al.*^46^ was benchmarked against PBE-D3 results of Comas-Vives^24^ (ESI† Fig. S1). A plane-wave energy cutoff of 450 Ry and a relative cutoff of 60 Ry were selected. Dispersion effects were accounted for using the D3BJ dispersion correction scheme of Grimme *et al.*^47^ To increase throughput, only during algorithmic saturation, the structures were partially optimized over 25 steps, resulting in forces generally below 1 × 10^−3^ Hartree Bohr^−1^. Otherwise, structures were optimized to tolerances of 4.5 × 10^−4^ and 3 × 10^−3^ Bohr for force and displacement, respectively.

Stability diagrams were constructed using atomistic thermodynamics.^48^ The Gibbs free energy (Δ*G*) was estimated using the equation

1 Δ*G* = *E*_SiO_2_,*ρ*_high__ − *E*_SiO_2_,surf_ − *n*_H_2_O_*μ*_H_2_O_(*T*,*P*_H_2_O_)

where *E*_SiO_2_,*ρ*_high__ is the electronic energy of the surface of the highest silanol density, *E*_SiO_2_,surf_ is the electronic energy of the silica surface for which Δ*G* is to be calculated, and *n*_H_2_O_ is the difference in the number of water molecules between the surfaces. *μ*_H_2_O_(*T*,*P*_H_2_O_) is the chemical potential of water which can be approximated by the ideal gas law through which the energy of the system at varying temperature (*T*) and partial pressure of water (*P*_H_2_O_) can be determined:

2 *μ*_H_2_O_(*T*,*P*_H_2_O_) = *E*_H_2_O_ + *μ*_H_2_O_(*T*,*P*°) + *k*_B_*T *ln(*P*_H_2_O_/*P*°)

where *E*_H_2_O_ is the electronic energy of water, and *μ*_H_2_O_(*T*,*P*°) is the chemical potential of water at a given temperature *T* and standard pressure *P*°. This term contains all contributions from rotations, vibrations, and ideal gas entropy at 1 atm. While this term can be calculated from first principles, the values stated in the JANAF thermodynamic tables^49^ were used instead. This method assumes that the phonon modes do not change during the functionalization process; even so, the error in this assumption is known to be smaller than the error inherent to DFT^50^ for a surface made with a perfect β-cristobalite. For evaluation of this assumption in the context of amorphous surfaces, see Section S2 in ESI.†

## 3. Results and discussion

**Fig. 5:**
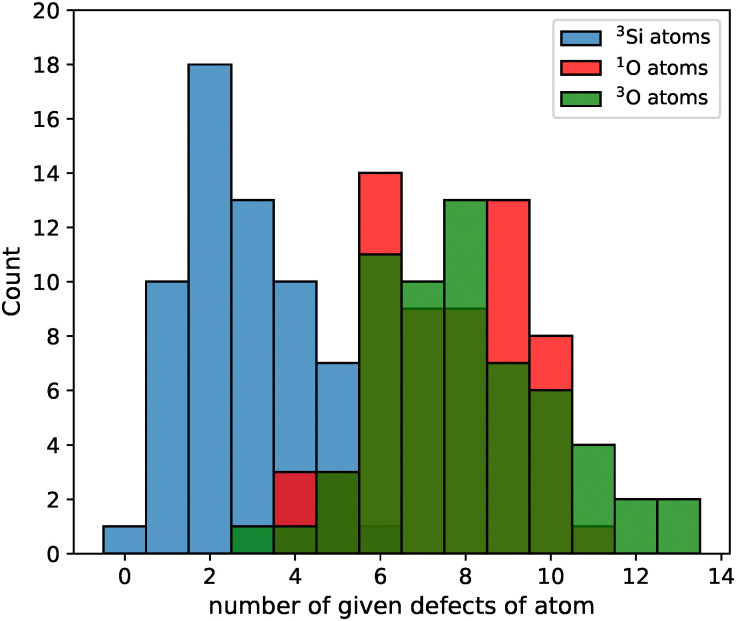
Count of a given number of defects occurring within a model for (blue) under-coordinated Si atoms (^3^Si), (red) under-coordinated O atoms (^1^O), and (green) over-coordinated O atoms (^3^O).

In preparation for algorithmic saturation, 60 slabs of different roughness had the coordination of each of their atoms calculated after the step “Relax initial structure” in Fig. 3. The saturation occurs at dangling atoms, namely singly bound O atoms (^1^O) and 3-coordinated Si atoms (^3^Si). Fig. 5 summarizes the distributions of such atoms among all slabs. One can see that the distributions of ^3^Si and ^1^O do not overlap substantially. Naively saturating all dangling bonds would therefore lead to models of net formal charge, non-physical considering the system we are aiming to model is a solid surface in contact with a gaseous phase. Thus, 3-coordinate O atoms (^3^O) were used to create additional ^3^Si to give formally-neutral slabs. The distributions of ^1^O and ^3^O overlap significantly, resulting in an excess of ^3^O that can be used. As the energetic effect of removing silanol groups cannot be known *a priori*, the impact of adding groups using one ^3^O over another was screened before choosing which structure to carry forward, in hopes of avoiding unnecessary additional instability to the model.

**Fig. 6:**
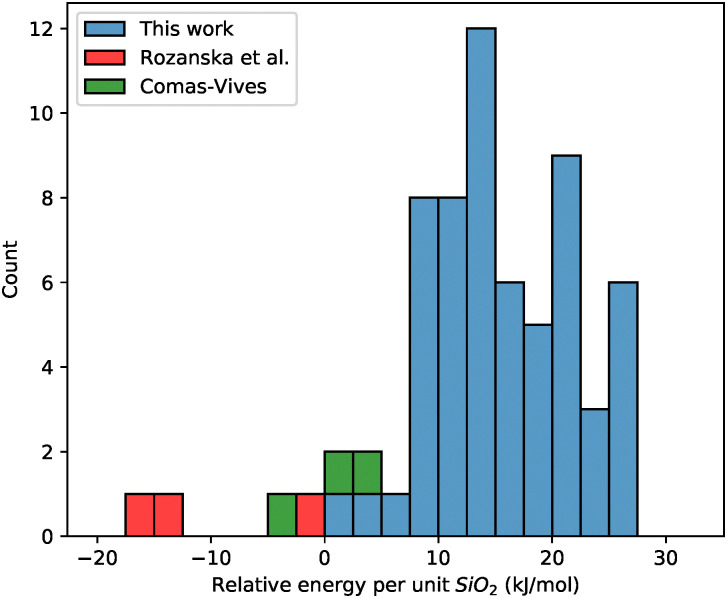
Count of models of given relative energy per unit SiO_2_ (reference states of β-cristobalite and H_2_O) of this work, Rozanska *et al.*,^50^ and Comas-Vives.^24^

The relative stabilities of the saturated slabs were analyzed. Energies were normalized per SiO_2_ unit relative to that of bulk β-cristobalite and H_2_O. For comparison, the readily available models of Comas-Vives^24^ and Rozanska *et al.*^50^ were relaxed using the same semi-empirical SCC-DFTB parameterization used during algorithmic saturation and the same relative stability was calculated, the results of which are summarized in Fig. 6. As expected, surface structures are less stable than bulk β-cristobalite, however, this is not true for all the previous models. All models made by Rozanska *et al.*^50^ and the model representing 2.0 OH nm^−2^ of Comas-Vives^24^ are more stable compared to the chosen reference states. We ascribe this over-stabilization to the asymmetric hydroxylation of the slabs. One side of those models remains at its initial silanol density and is not dehydroxylated in tandem with the other; thus, a network of hydrogen bonds remains. Our models are 1.5–27 kJ mol^−1^ (16 kJ mol^−1^ per SiO_2_ unit on average) less stable than the cristobalite reference and have two highly hydroxylated sides instead of one. These stability trends are in line with the expected lower intrinsic stability of the amorphous structures than that of the crystalline ones.

**Fig. 7:**
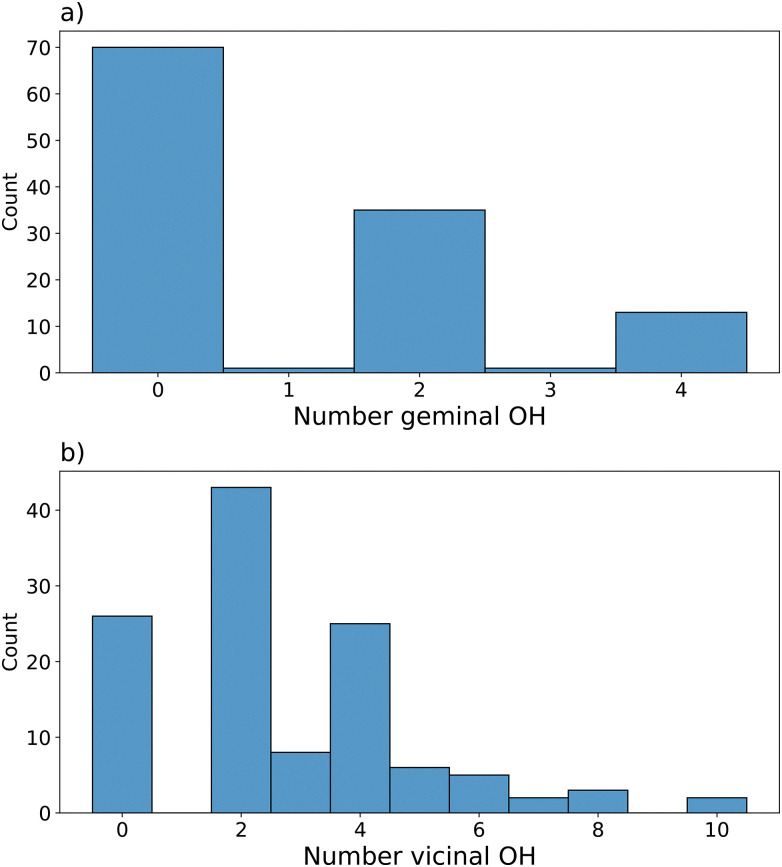
Count of surfaces within the data set with a given number of (a) –OH groups part of a geminal pair (b) vicinal silanol groups.

The algorithmic saturation gives rise to versatile silanol environments on the surface, which together with the variation in the roughness establish a wide range of chemical and topological compositions. Fig. 7a presents the probability of a given number of geminal groups forming on a surface using the presented approach. About 60% of surfaces had no geminal pairs, about 30% had one pair of geminal –OH groups and the remaining had two pairs. A surface with an odd number of geminal groups was found and is an artifact due to insufficient separation between the two surfaces of the slab. For simplicity, these models were not included in further analysis. Experimentally, it is postulated that 10% of silanol groups present on SiO_2−700_ are geminal.^13^ For the generated ensemble of surfaces, geminal silanol groups make up 14% of all SiOH.

Fig. 7b reports the number of surfaces with given numbers of vicinal silanol pairs. Surfaces of 0, 2, or 4 vicinal groups were predominantly generated with 2 groups accounting for 35% of the dataset and those of 0 or 4 accounting for 20%. Other periodic models for SiO_2−700_ have either one or no vicinal groups present. This is in line with the experimental findings that vicinal groups may be present even after SiO_2_ dehydroxylation at 800 °C.^13^ Furthermore, models with vicinal groups have been able to accurately reproduce experiments such as IR spectra^24,25^ or experimental silanol density as a function of temperature.^14^

**Fig. 8:**
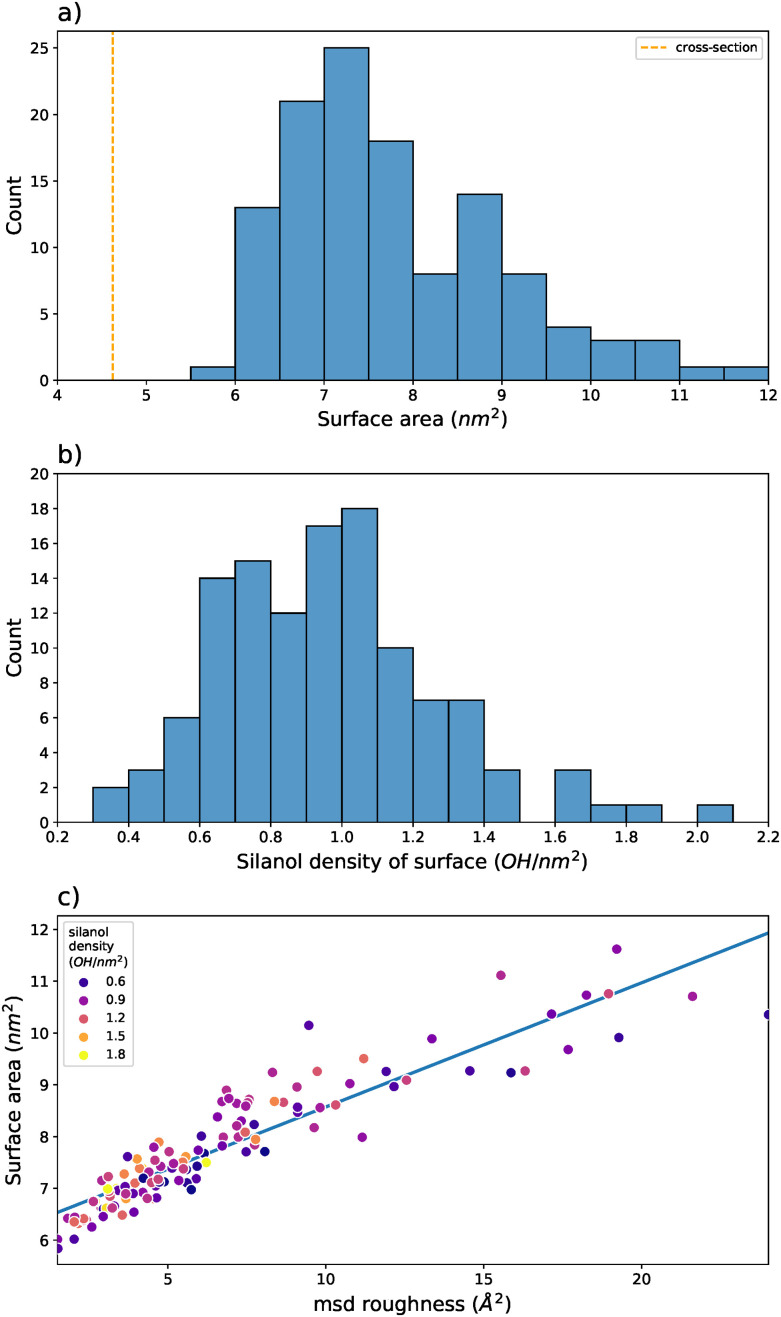
(a) Count of surfaces with a given solvent-accessible surface area. (b) Count of surfaces with given silanol densities. (c) Relationship between surface roughness and surface area with the densities of the points given through the color of the scatter.

To account for the generated models having increased roughness, the area of each surface, shown in Fig. 8a, was estimated to determine the silanol density more accurately. This resulted in values of surface area between 5.8 nm^2^ and 11.6 nm^2^, an average of 7.8 nm^2^. Using the previous methodology, the flat cleaving plane allows for the surface area to be estimated through the *xy* cross-sectional area of the periodic unit cell. This results in a surface area of 4.6 nm^2^ for all generated surfaces. In the worst case, this is a difference of a factor of 3. Compared to the average, this is underestimated by a factor of 1.7. Applying this method to the readily available models in literature, similar results were achieved (ESI† Table S2). In all cases, the calculated surface area of the dehydroxylated side of the model is approximately 50% larger than the cross-sectional area of the simulation box. For the models of Comas-Vives,^24^ this could be a fair approximation, for those of Rozanska *et al.*^50^ it could be as well. Visually inspecting the latter models, there are slight cavities that could lead to this greater-than-expected surface area. In any case, for models of amorphous nature, these estimates should be a sufficiently close approximation to the surface area of a model.

Based on the calculated surface areas, silanol densities ranging from 0.35 OH nm^−2^ to 2.00 OH nm^−2^ were generated, with an average density of 0.96 OH nm^−2^ (Fig. 8b). The range of silanol densities represents surfaces treated between 1000 °C and approximately 400 °C, respectively. The average almost perfectly matches the expected silanol density of SiO_2−700_.^13,14^ In line with prior studies, the roughness of our surfaces correlates strongly with their surface area (Fig. 8c).^32^ That said, there is no simple relationship between the resulting silanol density or roughness ultimately implying that the proposed method samples a wide range of topological and unique silanol group environments.

**Fig. 9:**
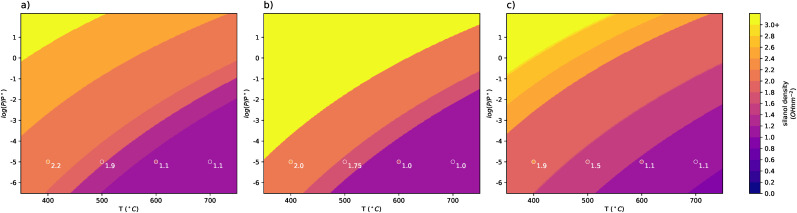
Stability diagrams as a function of log(*P*_H_2_O_/*P*°) and temperature (°C) constructed through further functionalizing a surface of (a) low, (b) medium, and (c) high surface roughness. Scatter points are colored according to experimental silanol density at thermodynamic conditions for 1.15 OH nm^−2^ (700 °C), 1.5 OH nm^−2^ (600 °C), 1.8 OH nm^−2^ (500 °C), 2.35 OH nm^−2^ (400 °C). Colors are chosen according to the color bar. Scatter points are labeled with the calculated stable silanol density for the surface at given thermodynamic conditions.

To establish whether the generated models could be representative of reaction conditions, atomistic thermodynamics was applied to calculate the relative stabilities. Surfaces of low, medium, and high roughness (surfaces noted in Table S3, ESI†) were further functionalized through the simple addition of H_2_O across siloxane bonds to get a range of silanol densities. Fig. 9 reports the silanol density of the most stable surface resulting from this additional functionalization as a function of *T* and *P*_H_2_O_. For the high-temperature dehydroxylation conditions employed for the generation of SiO_2−700_, the most stable medium- and high-roughness surfaces show SiOH densities of 1.1 OH nm^−2^ or 1.0 OH nm^−2^ in a perfect agreement with the experiment.^13^ Following the range of surface silanol densities generated, the stable surfaces at 400 °C, 500 °C, and 600 °C under vacuum are also denoted. Experimentally, they are determined to be 2.35 OH nm^−2^, 1.8 OH nm^−2^, and 1.5 OH nm^−2^, respectively.^13^ At 400, all three model surfaces are in line with these results, showing stable densities of 2.2 OH nm^−2^, 2.0 OH nm^−2^, and 1.9 OH nm^−2^, for low, medium, and high roughness. Similar results are also seen for 500 °C. At 600 °C, the stable surface is the same as that stable at 700 °C.

As the number of Si–O bonds that can be randomly broken for any one addition H_2_O is so large within the ensemble, resulting in errors in stability diagrams due to non-exhaustive search of configurational space and, thus, neglect of configurational entropy may be significant. This should be the most noticeable at higher silanol densities and still difficult to quantify even at low silanol densities. The process of further functionalizing the surfaces was repeated to assess whether results can be replicated. This was done for the high and low roughness surfaces (ESI† Fig. S3 and S4). As silanol density increases, deviations in the stable densities at given *T* and *P*_H_2_O_ do indeed arise. For the low roughness surface, the stable surface at 400 °C is different between each iteration is different. For high roughness, slight deviation even at 600 °C. Still, the surfaces of highest stability at 700 °C remain the same across iterations, specifically those around 1.1 OH nm^−2^.

## 4. Conclusion

Within this study, a systematic and general approach for the creation of period slab models of amorphous materials is proposed and was used to generate 120 surfaces. These models will help simulate and understand the reactivity of amorphous materials. Dehydroxylated amorphous silica was chosen as a representative example, where we were able to generate 120 surfaces with a variety of roughness, silanol compositions, and silanol densities, aiming to represent the material activated at 700 °C.

The silanol densities of the surfaces were on average 0.96 OH nm^−2^ after estimating the surface area through a polygon mesh of the solvent-accessible surface area of Kr. Without calculating this estimate, surface areas would have errors around a factor of 1.7, showing that the previous method of using the *xy*-cross-sectional area quickly underestimates this property even after the smallest amount of surface roughness is introduced. Not only is the initial silanol density almost exactly that of SiO_2−700_, but the numbers of geminal and vicinal silanol groups are in good agreement with experimental results. While vicinal groups are occasionally in excess, the removal of 1 or 2 H_2_O molecules is minimal compared to the number that has to be removed following older methodologies.

The plausibility of the models was checked by comparing them against experimentally measured silanol density at given *T* and *P*_H_2_O_. For three surfaces of varying roughness, silanol groups were naively added to the surface and the preferred stability was evaluated using atomistic thermodynamics. At 700 °C in vacuum, the preferred silanol density of the model surfaces was between 1.0 OH nm^−2^ and 1.1 OH nm^−2^. Decreasing the temperature to 600 °C, the preferred stability did not change. Moving to 500 °C and subsequently 400 °C, the preferred stability followed experimental results of 1.8 OH nm^−2^ and 2.35 OH nm^−2^. Repeating the process for the same models twice more, results remained consistent. Thus, with not only surface silanol characteristics but also thermodynamic properties being accurate for that of SiO_2−700_, this ground-up method can be a more computationally efficient alternative for the generation of highly dehydroxylated periodic slabs of amorphous silica. The efficiency of this method, and algorithm, allows one to generate numerous surface models for an amorphous material at a minimal computational cost.

## Author contributions

M. P. Klein: investigation; acquire data; coding; formal analysis of data; writing original draft; writing – review & editing A. A. Kolganov: conceptualization, supervision, writing – review & editing E. A. Pidko: writing – review & editing, funding acquisition.

## Conflicts of interest

There are no conflicts to declare.

## Supplementary Material

CP-027-D5CP01570G-s001

CP-027-D5CP01570G-s002

## Data Availability

All supporting data, including trajectories, output files, surface structures, and code, will be available at 4TU. Research Data at DOI 10.4121/b8b88018-26a2-41cf-b5cc-10627cb94c0 at the time of publication.
