# Supplementary material for: Ground-up generation of periodic slab models of dehydroxylated amorphous silica of varying roughness
Source: Phys Chem Chem Phys. 2025 Jun 26;27(28):15196–203. doi: 10.1039/d5cp01570g (PMC12232287; doi:10.1039/d5cp01570g)
Supplement: CP-027-D5CP01570G-s001 [file CP-027-D5CP01570G-s001.pdf]

Supplementary Information (SI) for Physical Chemistry Chemical Physics.
This journal is © the Owner Societies 2025

ESI

S1

Table S1: Forcefield parameters used for classical molecular dyanmics simulations. Buckingham parameter of van Beest, Kramer, and van Santen.[1] Lennard-Jones paramters gotten from Wimalasiri et al.[2]

	Buckingham parameters			Atomic charges		Lennard-Jones parameters	
i-j	$A_{ij}(eV)$	$b_{ij}(\mathring{A}^{-1})$	$c_{ij}(eV\ \mathring{A}^6)$	site i	$q_e(e)$	$\epsilon_{ij}(eV)$	$\sigma_{ij}(\mathring{A})$
Si-Si	0	0	0	Si	2.4	0	0
Si-O	1388.8	2.7600	175.00	O	-1.2	2.6	1.6
Si-O	18044	4.8722	133.54	-	-	2.0	1.2

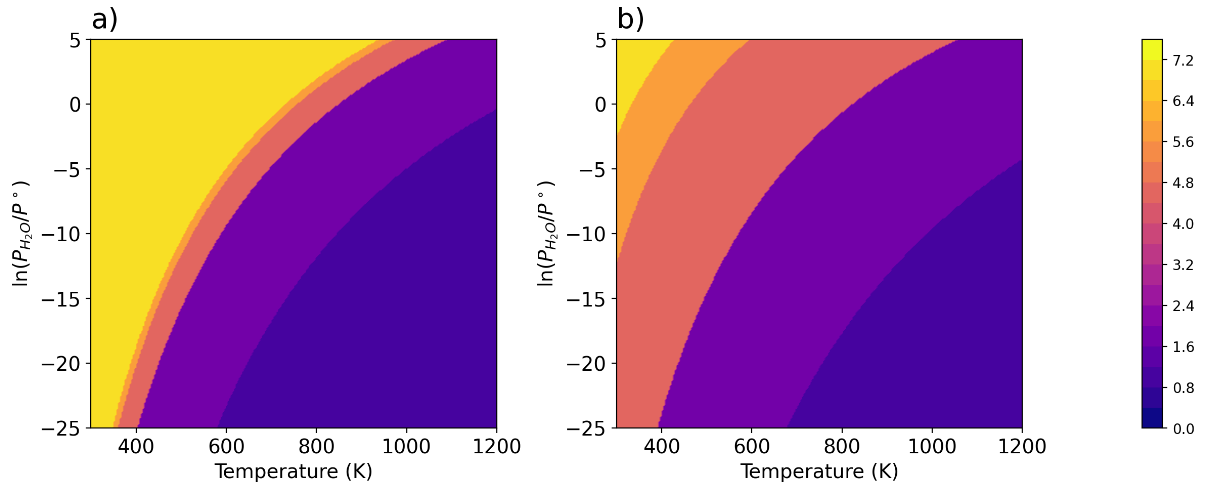

Fig. S1: Stability diagram as a function of $\ln(P_{H_2O}/P^{\circ})$ and temperature (K) constructed from the model of Comas-Vives[3] optimized using a) DFT, as described by Comas-Vives[3] and b) SCC-DF

S2

Previous work showed that neglecting vibrational contributions to free energy is on the order of magnitude of the expected error related to DFT.[4] However, these calculations are for a slab made using β-cristobalite. When applying this assumption to an amorphous surface, the rearrangement could be great enough such that the assumption that phonon modes remain similar no longer holds. To assess the error related to neglected phonon calculations, full phonon mode calculations for one of the further saturated surfaces were done and the phase diagram was recalculated. This was done in the CP2K software package using full boundary conditions, displacements of 0.001 Å, and a SCF convergence criteria of 1 $\times 10^{-9}$. The vibrational entropy (S_ν) and enthalpy (H_ν) were calculated according to:

$$S_\nu = \sum_i \left(\frac{h\nu_i}{k_B T} \frac{1}{e^{-\frac{h\nu_i}{k_B T}} - 1} - \ln(1 - e^{-\frac{h\nu_i}{k_B T}}) \right) \tag{1}$$

and

$$H_\nu = \sum_i \frac{h\nu_i}{k_B T} \left(\frac{1}{2} + \frac{1}{e^{-\frac{h\nu_i}{k_B T}} - 1} \right) \tag{2}$$

Here ν_i is every calculated vibrational frequency i in units of s^{-1}, k_B and h are Boltzmann's and Planck's constants. T represents temperature.

For this assessment, the surface of high roughness was chosen as it was reasoned to be the most likely to undergo substantial rearrangements. The new stability diagram can be found in Figure S2a, with the other diagram (Figure S2b) corresponding to Figure 9c in the main text. What can be observed is that low silanol density surfaces are stabilized and at higher partial pressures of water, surfaces of greater silanol densities are stabilized. Still, the surface most stable at the conditions of primary interest, 700 °C and vacuum, remains the same.

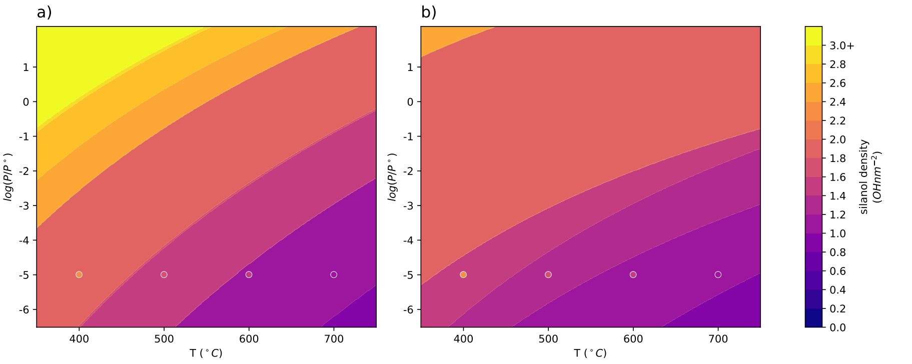

Fig. S2: Stability diagram as a function of $\log(P_{H_2O}/P^\circ)$ and temperature (°C) comparing a) neglecting vibrational contributions to free energy and b) full vibrational contribution to free energy.

Table S2: Surface area (SA), roughness (msd), number of -OH groups (num OH), silanol density (density), number of geminal -OH groups (num gem), number of vicinal -OH groups (num vic), and number of isolated silanol groups (num iso) of the top and bottom surface of all models generated

Model source	SiOH density (OH nm^{-2})	cross-sectional area (nm^2)	calculated surface area (nm^2)
Comas-Vives[3]	2.0	4.57	6.75
Comas-Vives[3]	1.6	4.57	6.36
Comas-Vives[3]	1.1	4.57	6.34
Rozanska et al.[4]	1.42	3.23	4.31
Rozanska et al.[4]	1.86	2.16	2.99
Rozanska et al.[4]	1.75	2.29	3.33

S3

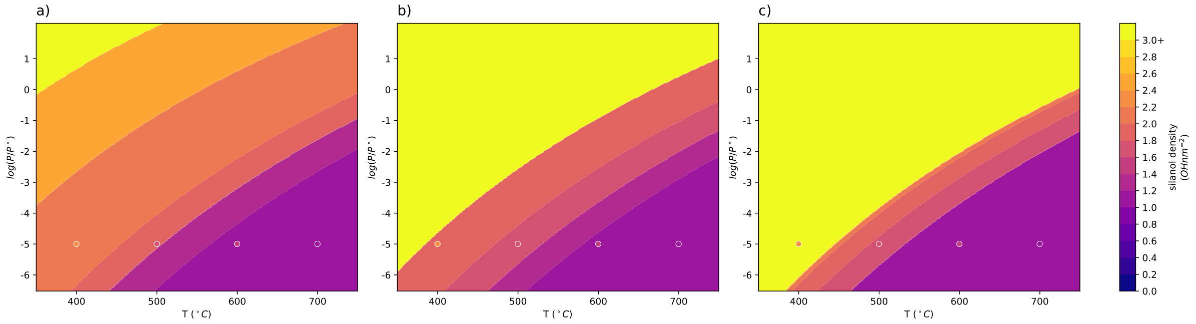

Fig. S3: Stability diagram as a function of $\log(P_{H_2O}/P^{\circ})$ and temperature (K) for the surface of low roughness for a) initial iteration b) repetition 1 c) repetition 2

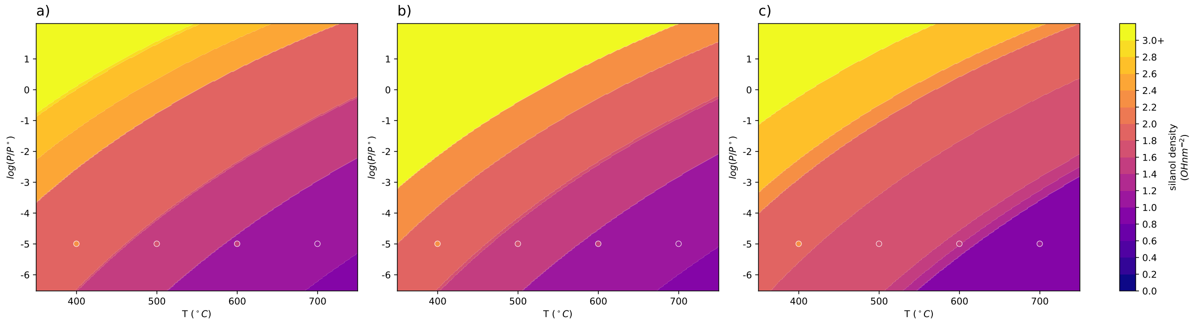

Fig. S4: Stability diagram as a function of $\log(P_{H_2O}/P^{\circ})$ and temperature (K) for the surface of high roughness for a) initial iteration b) repetition 1 c) repetition 2

Table S3: Surface area (SA), roughness (msd), number of -OH groups (num OH), silanol density (density), number of geminal -OH groups (num gem), number of vicinal -OH groups (num vic), and number of isolated silanol groups (num iso) of the top and bottom surface of all models generated

model number[a]	Top surface							Bottom surface						
	SA (Å^2)	msd (Å^2)	num OH	density (OH nm^{-2})	num gem	num vic	num iso	SA (Å^2)	msd (Å^2)	num OH	density (OH nm^{-2})	num gem	num vic	num iso
0	602	1.51	5	0.831	0	2	3	675	2.70	11	1.630	0	5	6
1	856	9.82	8	0.935	0	4	4	927	16.30	10	1.080	4	2	4
2	700	3.38	12	1.710	0	8	4	692	4.21	6	0.867	0	4	2
3	663	2.89	7	1.060	0	2	5	739	5.13	5	0.676	0	0	5
4	789	4.72	11	1.390	4	0	7	799	11.20	7	0.876	4	3	0
5	866	8.66	10	1.150	0	7	3	909	12.60	10	1.100	4	4	2
6	738	4.20	12	1.630	2	5	5	632	2.17	8	1.260	0	8	0
7	784	7.75	8	1.020	4	2	2	968	17.70	8	0.826	2	2	4
8	767	6.15	3	0.391	0	0	3	690	3.90	5	0.725	0	2	3
9	639	2.43	5	0.782	0	0	5	639	2.34	7	1.100	0	2	5
10	799	7.23	8	1.000	1	2	5	1071	21.60	10	0.934	3	2	5
11	750	6.22	14	1.870	2	7	5	989	13.40	8	0.809	0	4	4
12	680	3.67	10	1.470	0	4	6	821	7.18	8	0.975	2	2	4
13	584	1.52	4	0.685	0	2	2	699	3.09	14	2.000	2	4	8
14	1162	19.20	10	0.861	2	6	2	774	5.96	6	0.775	0	4	2
15	761	5.57	11	1.440	0	8	3	715	2.91	7	0.979	0	4	3
16	751	5.47	10	1.330	0	10	0	817	9.63	8	0.979	0	3	5
17	872	7.57	9	1.030	4	0	5	801	6.06	5	0.624	2	0	3
18	706	4.63	7	0.992	0	2	5	666	3.31	5	0.751	0	2	3
19[b]	1076	19.00	12	1.120	4	5	3	1037	17.10	8	0.772	2	2	4
20	710	3.96	9	1.270	0	5	4	704	3.65	5	0.711	0	2	3
21	660	2.96	4	0.606	0	2	2	874	6.93	8	0.916	2	2	4
22	847	9.11	6	0.708	0	3	3	723	3.11	8	1.110	2	2	4
23	864	7.18	8	0.926	2	4	2	642	2.33	8	1.250	0	4	4
24	779	4.56	7	0.898	0	4	3	698	5.74	3	0.430	0	0	3
25	738	4.11	10	1.350	2	3	5	648	3.56	8	1.230	0	2	6
26	868	6.71	8	0.922	4	2	2	743	5.93	4	0.538	0	0	4
27	857	9.11	6	0.700	2	2	2	644	2.05	6	0.931	0	2	4
28	861	10.30	10	1.160	2	2	6	738	5.49	8	1.080	2	4	2
29[c]	736	5.59	4	0.543	0	0	4	859	7.47	8	0.931	2	0	6
30	809	7.45	10	1.240	0	6	4	715	5.35	6	0.839	2	0	4
31	771	5.04	8	1.040	0	4	4	635	2.04	8	1.260	2	2	4
32[d]	923	15.90	5	0.542	0	0	5	771	8.07	3	0.389	0	0	3
33	713	4.88	3	0.421	0	0	3	662	3.06	11	1.660	0	6	5
34	719	5.90	5	0.696	0	0	5	662	3.24	7	1.060	0	2	5
35	838	6.57	7	0.835	2	0	5	823	7.73	5	0.607	2	2	1
36	705	4.61	5	0.709	2	0	3	761	3.72	5	0.657	0	2	3
37	731	4.40	7	0.957	0	4	3	795	7.78	11	1.380	0	10	1
38	696	3.43	5	0.718	2	0	3	682	4.65	5	0.733	0	0	5
39	896	9.09	9	1.000	2	4	3	926	9.73	11	1.190	4	4	3
40	927	14.60	6	0.647	2	2	2	991	19.30	6	0.605	0	4	2
41	720	4.22	3	0.417	0	0	3	654	3.93	5	0.764	0	4	1
42	865	7.52	9	1.040	2	2	5	743	4.77	7	0.943	0	2	5
43	868	8.37	12	1.380	0	5	7	799	6.75	8	1.000	0	2	6
44	685	3.17	8	1.170	0	5	3	712	4.73	4	0.561	0	0	4
45	902	10.80	8	0.887	2	0	6	897	12.20	6	0.669	0	0	6
46	694	3.13	8	1.150	2	3	3	754	4.59	8	1.060	2	2	4
47	711	4.49	8	1.120	0	3	5	951	11.20	12	1.260	4	4	4
48	889	6.86	9	1.010	2	4	3	1073	18.30	9	0.839	2	2	5
49	680	4.55	9	1.320	2	2	5	690	3.67	7	1.020	2	2	3
50	1015	9.46	7	0.690	2	2	3	1111	15.50	11	0.990	0	4	7
51	642	1.83	6	0.934	0	4	2	718	4.70	8	1.110	0	6	2
52	771	7.48	5	0.649	0	0	5	748	5.18	7	0.936	0	6	1
53	639	2.42	7	1.100	0	0	7	646	2.95	5	0.774	0	4	1
54	711	5.60	4	0.563	0	3	1	782	6.72	6	0.767	0	2	4
55	728	3.62	10	1.370	2	3	5	602	2.03	4	0.664	0	2	2
56	625	2.59	5	0.799	0	2	3	681	4.35	7	1.030	0	4	3
57	757	4.03	11	1.450	4	4	3	675	2.64	7	1.040	0	2	5
58	830	7.32	7	0.843	2	2	3	924	8.31	9	0.974	4	2	3
59	926	11.90	6	0.648	4	0	2	1035	24.00	6	0.579			

[a] Models numbered according to the naming of provided structures and optimization trajectories.
[b] Top surface of this model used to represent low roughness
[c] Top surface of this model used to represent medium roughness
[d] Top surface of this model used to represent high roughness

References

[1] B. W. H. van Beest, G. J. Kramer and R. A. van Santen, *Phys. Rev. Lett.*, 1990, **64**, 1955–1958.

[2] P. N. Wimalasiri, N. P. Nguyen, H. S. Senanayake, B. B. Laird and W. H. Thompson, *The Journal of Physical Chemistry C*, 2021, **125**, 23418–23434.

[3] A. Comas-Vives, *Phys. Chem. Chem. Phys.*, 2016, **18**, 7475–7482.

[4] X. Rozanska, F. Delbecq and P. Sautet, *Phys. Chem. Chem. Phys.*, 2010, **12**, 14930–14940.
